# ‘Suicide rates in Crete, Greece during the economic crisis: the effect of age, gender, unemployment and mental health service provision’

**DOI:** 10.1186/s12888-018-1931-4

**Published:** 2018-11-01

**Authors:** Maria Basta, Alexandros Vgontzas, Anastasia Kastanaki, Manolis Michalodimitrakis, Katerina Kanaki, Katerina Koutra, Maria Anastasaki, Panagiotis Simos

**Affiliations:** 1 Department of Psychiatry, University Hospital of Heraklion, Voutes,Heraklion, 71110 Crete, Greece; 2 Department of Forensic Science, School of Medicine, University of Crete, Voutes, Heraklion, 71003 Crete, Greece; 3 Department of Psychology, School of Social Sciences, University of Crete, Gallos, Rethymnon, 74100 Crete, Greece

**Keywords:** Suicides, Gender, Health services, Economic crisis, Greece

## Abstract

**Background:**

Recently, suicides in Greece have drawn national and international interest due to the current economic crisis. According to published reports, suicides in Greece have increased up to 40% and Crete has been highlighted as an area with the sharpest increase.

**Aim:**

To investigate the suicide mortality rates in Crete between 1999 and 2013 and their association with the economic crisis.

**Methods:**

Data on suicides were selected from the Department of Forensic Medicine files of the University of Crete.

**Results:**

Our analysis showed that (1) Crete, has the highest suicide mortality rate in Greece, however no significant increase was observed between 1999 and 2013, (2) there were opposing trends between men and women, with women showing a decrease whereas men showed an increase in that period, (3) there was a significant increase of suicides in middle-aged men (40–64 yrs) and elderly, although the highest unemployment rates were observed in young men and women, and (4) finally, there was a regional shift of suicides with a significant decrease in Western Crete and a significant increase in Eastern Crete.

**Conclusions:**

Although, Crete has the highest suicide mortality rates in Greece, we did not observe an overall increase during the last 15 years, including the period of economic crisis. Furthermore, there was an increase in middle-aged and elderly men, whereas young men and women showed oppositional trends during the years of austerity. This may be related to the culturally different expectations for the two genders, as well as that younger individuals may find refuge to either strong family ties or by immigrating abroad. Finally, the relative increase of suicides in Eastern Crete may be explained by factors, such as the lack of community mental health services in that area.

## Background

For the last several years suicides in Greece have drawn national and international interest due to the current economic crisis, which has engulfed Europe since 2008. This period coincided with the implementation of fiscal austerity, increase of unemployment rates and negative economic growth, and had severe adverse effects on various aspects of people’s daily life and probably on their mental health. Studies in European Union countries conducted during the recent economic crisis period have found associations between suicide mortality rates (SMRs) and unemployment [1]. In Greece, according to published reports, SMRs have been on a remarkable upward trend up to 40% [2–4]. In 2011 a brief report in Lancet suggested a 40% rise in suicides in the first half of 2011 compared to 2010 [5]. Kontaxakis et al. reported that in the decade between 2001 and 2011 SMRs increased by 38.4%, with women showing the highest rise (69.6% as compared to 33.1% among men) [3]. In a similar vein, another recent study reported a 35% increase in suicides between 2010 and 2012, with unemployment being significantly associated with suicide mortality, especially among men of working-age, which coincided with austerity measures [4]. In another study it was suggested that fiscal austerity measures and negative economic growth were related to significantly increased male SMRs, whereas fiscal austerity affected mostly the population between 45 and 89 years of age [6]. Conversely, another long-term study from North Greece found no associations between SMRs and unemployment [7].

Based on a previous report, Crete displayed the highest suicide rate in Greece between 1999 and 2010 [8] and the lay press have designated the island of Crete as the area with the sharpest increase of suicides in Greece during the first years of the crisis. It should be noted, however, that although suicide mortality rates are known to fluctuate yearly, requiring relatively longer study periods, the aforementioned issue has not been systematically addressed using data from subsequent years (http://agonaskritis.gr/οι-αυτοκτονίες-στην-κρήτη; https://www.neakriti.gr/article/eidiseis/989417/kriti-tragiki-prwtia-stis-aytoktonies/).

The present study included data from 1999 to 2013 in order (a) to compare suicide mortality rates before and during the economic crisis, and (b) to examine short and long term effects of the crisis. Furthermore, we assessed the association between specific sociodemographic parameters (i.e. gender, age, unemployment rates and availability of mental health services in Western vs. Eastern Crete) on differences in SMRs between the two time periods. Our initial hypothesis was that suicides have increased during the crisis period, and that this increase is associated with economic indices, i.e. unemployment, and is influenced by the availability of mental health services.

## Methods

### Study design

A retrospective study was undertaken reviewing all suicide cases in Crete between January 1, 1999 and December 31, 2013. Crete is the largest of the Greek islands with a population of 623,065 in the 2011 by the Hellenic Statistical Authority (ELSTAT) census. The island of Crete is divided into four prefectures, which from west to east are the following: Chania, Rethymnon, Heraklion and Lasithi. Data on the suicide fatalities in Crete were obtained from the Department of Forensic Sciences, Faculty of Medicine, University of Crete, Greece. A total of 651 cases were registered during this period. National suicide data were provided by ELSTAT, an independent, national authority in Greece that follows European and international standards of data collection and statistical analyses. The study has been approved by the IRB Committee of the University of Crete, School of Medicine.

### Data analysis

Suicide mortality rates (SMRs) per 100,000 inhabitants were calculated for each calendar year as well as age-adjusted SMRs (based on the 2001 population census) with the direct method. Region-specific (i.e., Western and Eastern Crete) and gender-specific rates were also calculated. Previous research has shown that the association of suicide with socio-economic variables varies with gender and age [9]. Thus, we also stratified the sample into meaningful age-subgroups to depict those who had recently entered the labor market (< 40 years old), those who had been in the workforce longer (40–64 years old) and finally those at retirement age (> 64 years) and calculated age- by gender-specific SMRs for each subgroup. Yearly trends in SMRs were modeled using Poisson Regression. Model fit was assessed using the Wald Chi-square test at *p* = 0.05. Further the association between suicide and unemployment rates as a function of Age Group was examined using Generalized Linear Model analysis. All analyses were performed with IBM-SPSS v. 20.

Given that prior studies have raised the issue of potential misclassification of suicide as a cause of death in numerous countries, including Greece (mostly due to religious and other reasons) [10–12], more recent studies took measures to eliminate this possibility [2]. Following this suggestion and in order to investigate the potential bias of suicide misclassification in our analyses, we performed a series of sensitivity analyses including a comparison of official suicide mortality data from ELSTAT with validated coroner death certificate data for the same suicides in the island of Crete.

## Results

During the 15 years examined a total of 651 suicides were recorded in Crete, 527 (80.9%) were committed by men and 124 (19.1%) by women. Standardized SMRs were nearly four times higher among men than among women (11.69 vs. 2.75 per 100.000 residents; Wald’s *X*^*2*^[1] = 6.94, *p* = 0.007).

### Suicide mortality rates in Crete vs. Greece

The first significant finding of this study is that SMRs in Crete are higher compared to the SMRs in Greece (see Table 1). This finding is verified based both on ELSTAT (Hellenic Statistical Authority) and the Archives of Forensic Medicine [Forensic Medicine, University of Crete (AFM)]. Specifically, based on ELSTAT data, the mean yearly SMR between 1999 and 2013 for Crete vs. Greece was 5.2 vs. 2.4 per 100,000 for the total group (Wald’s *X*^*2*^[1] = 11.43, *p* = 0.001), 10.8 vs. 5.3 for men (Wald’s *X*^*2*^[1] = 7.79, *p* = 0.005) and 2.5 vs. 1.1 for women (Wald’s *X*^*2*^[1] = 5.84, *p* = 0.0016), respectively. The men/women ratio was similar in both groups: 4.03 in Crete vs. 4.7 in Greece. In Crete there was a significant linear rise in this ratio during the study period (R^2^ = 0.577, *p* = 0.001).

**Table 1:** Standardized annual suicide mortality rates in Crete and Greece for men and women between 1999 and 2013

Crete			Greece			
Year	Men	Women	Total	Men	Women	Total
1999	10.43	3.71	7.13	5.13	1.35	3.19
2000	14.38	5.10	9.73	5.07	1.35	3.16
2001	9.39	1.59	5.44	4.81	0.77	2.75
2002	8.89	2.42	5.68	4.22	1.00	2.56
2003	11.11	2.40	6.83	5.13	1.12	3.07
2004	6.62	2.54	4.60	4.62	1.04	2.78
2005	11.98	2.71	7.27	5.07	1.25	3.10
2006	10.36	2.56	6.50	5.09	1.11	3.05
2007	8.59	1.84	5.17	4.34	0.91	2.60
2008	11.33	2.40	6.84	4.78	0.99	2.85
2009	11.18	2.69	6.92	5.23	0.86	3.02
2010	8.78	1.14	4.94	5.17	0.62	2.86
2011	12.6	2.26	7.46	6.17	1.32	3.71
2012	13.95	1.69	7.85	7.83	1.65	4.70
2013	12.66	2.38	7.57	8.01	1.96	4.93
Mean	10.81	2.49	6.66	5.38	1.15	3.10

The high SMR in Crete vs. Greece was verified by the data provided by the Archives of Forensic Medicine, although the number of suicides per year was higher compared to the numbers given by ELSTAT throughout all years by 8.2%.

### Suicide mortality rates in Crete during the period of the economic crisis: Gender effect

**Fig. 1:**
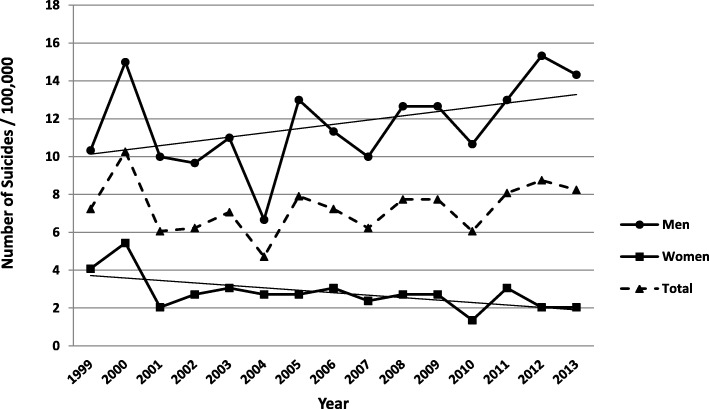
Yearly trends in standardized suicide mortality rates for the entire population of Crete (dashed line), men (solid line, circles) and women (solid line, squares) between 1999 and 2013

The second important finding of this study is that overall suicidal rates in Crete were rather stable during the 15-year study period, including the 2008–2013 interval of the financial crisis in Greece. Specifically, the Poisson regression analysis revealed that for every year during the study period there was a slight increase of 0.010 suicides per 100,000 residents, which failed to reach significance (95% CI =0.980 to 1.041, *p* = 0.5). Given that this effect was superseded by a Year by Gender interaction (Wald’s *X*^*2*^[1] = 4.96, *p* = 0.05), yearly suicide trends were assessed separately for men and women. As shown in Fig. 1, the SMR among men in Crete between 1999 and 2013 rose at an average annual rate of 0.025 instances per 100,000 residents, a trend that failed to reach significance (*p* = 0.16). Conversely, the SMR among women displayed a decreasing trend averaging 0.50 cases per year per 100,000 residents also failing to reach significance (*p* = 0.15).

Systematic differences in SMRs as a function of age were also assessed given a significant Age Group (< 40, 40–64, and > 64 years) by Year interaction among men (Wald’s *X*^*2*^[1] = 12.19, *p* = 0.002). Specifically, SMRs displayed average yearly increases of 0.48 and 0.31 suicides per year per 100,000 residents in the 40–64 year group (95% CI = 1.019 to 1.078, *p* = 0.001) and the > 64 year group, respectively (95% CI = 1.005 to 1.057, *p* = 0.018) (Table 2). In women annual trends in SMRs did not vary significantly across Age Groups (Age Group by Year interaction: *p* = 0.1).

**Table 2:** Standardized annual suicide mortality rates in Crete by gender and age group between 1999 and 2013

Age Group						
	Men			Women		
Year	18–39 yrs	40–64 yrs	> 64 yrs	18–39 yrs	40–64 yrs	> 64 yrs
1999	7.00	18.95	6.78	1.91	3.57	11.33
2000	12.25	13.02	29.36	3.18	10.71	3.78
2001	2.92	16.58	24.84	0.00	2.38	7.55
2002	8.17	10.66	13.55	1.27	3.57	5.66
2003	6.42	17.76	15.81	1.27	2.38	9.44
2004	5.25	8.29	9.03	3.18	1.19	3.78
2005	5.83	17.76	31.62	1.91	3.57	3.78
2006	8.17	14.21	18.07	0.64	5.95	5.66
2007	3.50	13.02	29.36	1.27	2.38	5.66
2008	4.67	21.31	27.10	0.00	7.14	3.78
2009	4.67	21.31	27.10	0.64	8.33	0.00
2010	7.00	11.84	22.59	0.00	3.57	1.89
2011	5.83	26.05	15.81	1.27	3.57	7.55
2012	5.83	28.42	27.10	1.27	3.57	1.89
2013	4.08	28.42	27.10	0.00	4.76	3.78
Mean	6.11	17.84	21.68	1.19	4.44	5.03

### Suicide mortality rates in Crete during the period of the economic crisis: Age effect

Another interesting finding is that SMRs in younger people of both genders were lower compared to those of middle-aged and retirement-age people. In both genders the Age Group main effect was significant (Men: Wald’s *X*^*2*^[2] = 11.09, *p* = 0.004; Women: Wald’s *X*^*2*^[2] = 9.02, *p* = 0.01) indicating significantly lower overall SMRs in the youngest age groups (6.01 in men and 1.03 suicides in women per 100,000 residents) as compared to both the 40–64 year group (17.44 in men and 4.53 suicides in women per 100,000 residents; *p* < 0.007) and to the > 64 year group (21.54 in men and 4.96 suicides in women per 100,000 residents; *p* < 0.007).

**Fig. 2:**
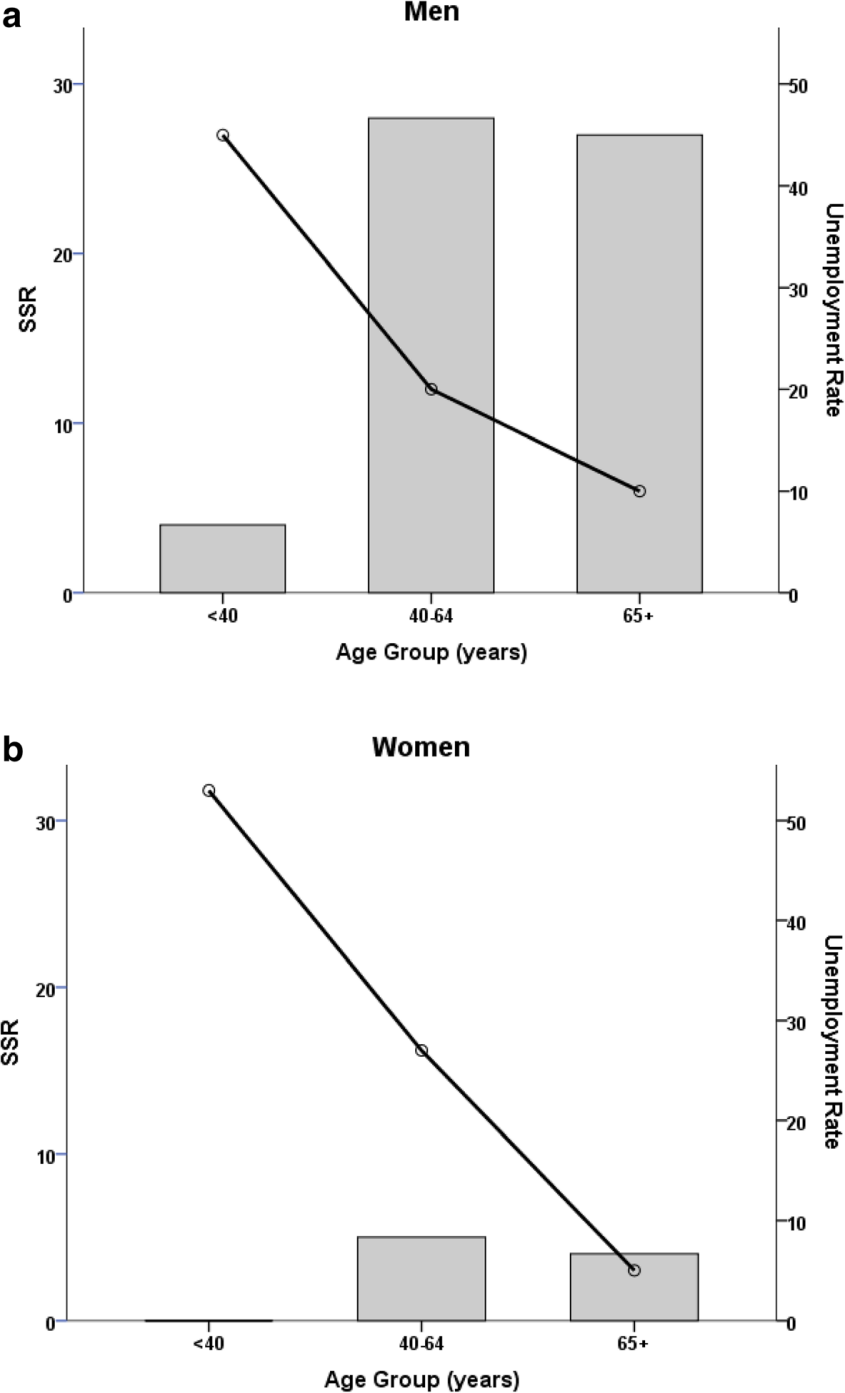
Average suicide mortality rates ([SSR]; per 100,000 residents; bars) and average unemployment rates (lines) for three age groups in Crete (2013 data)

To assess the hypothesis that suicides may be associated with the economic crisis in Greece, we examined the relation between suicide and unemployment rates in Greece during 2013—i.e., the year with the highest unemployment rate and the longest, cumulative crisis impact, among the years of the crisis examined in our study—based on data given by ELSTAT (http://www.statistics.gr/en/home/) (as an indicator of the impact of the economic crisis) stratified by gender and age group (Fig. 2). In men the significant *positive* association between age and SMR (Wald’s *X*^*2*^[2] = 13.88, *p* = 0.001) was paralleled by a significant *negative* association with unemployment rates (Wald’s *X*^*2*^[2] = 22.75, *p* < 0.0001). In women, whereas SMR did not vary significantly with age (*p* > 0.3), unemployment rates were also negatively associated with age (Wald’s *X*^*2*^[2] = 29.55, *p* < 0.0001). Specifically, younger men (< 40 years of age) had the highest unemployment rates (45%) and the lowest SMR (4.1 per 100,000 residents) among all age groups. Conversely, men aged 40–64 years had lower unemployment rates (20%) but presented the highest SMR among all age groups (28.42 per 100,000 residents). Similarly, younger women (aged < 40 years) had the highest unemployment rates (53%) and the lowest SMR (0 cases) among all age groups, while middle-aged women (aged 40–64 yrs) had lower unemployment rates (27%) and higher SMR (4.8 per 100,000 residents).

### Suicide mortality rates in Crete during the period of the economic crisis: Region effect

**Fig. 3:**
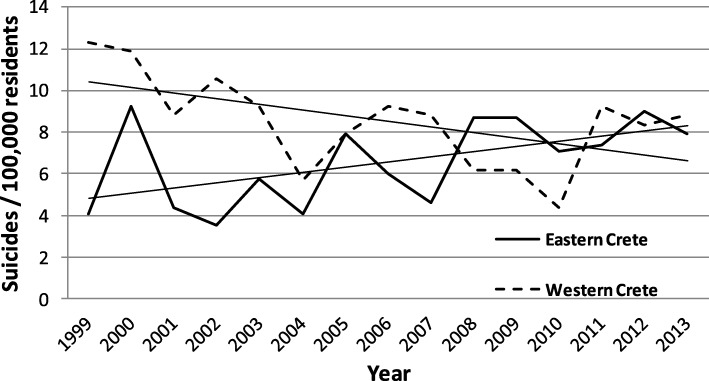
Yearly trends in standardized suicide mortality rates in Eastern (solid line) and Western Crete (dashed line) between 1999 and 2013

Finally, SMRs were analyzed based on the region where they took place. Crete was divided into two main regions: Western Crete (Chania and Rethymno prefectures), and Eastern Crete (Heraklion and Lasithi prefectures). The division “western” and “eastern” was based on the fact that after the closure of the only psychiatric hospital, that was located in Chania (western Crete), most of the new community mental health services were created on this area of the island, whereas there was a scarcity of mental health services in the east part. As already stated, one of the objectives of the study was to assess the influence of the availability of mental health services on SMR. Poisson regression results on SMR values revealed a significant Region main effect (Wald *X*^*2*^[1] = 8.42, *p* = 0.004), indicating overall higher SMRs in Western Crete, and a Region by Year interaction (Wald’s *X*^*2*^[1] = 5.43, *p* < 0.02). Figure 3 shows SMR values in Eastern and Western Crete. In Eastern Crete, the annual SMR rose significantly between 1999 and 2013 at an average rate of 0.39 suicides per 100,000 residents (95% CI = 1.014 to 1.064, *p* = 0.002). Conversely, in Western Crete there was a significant reduction in the total number of suicides at an average annual rate of 0.34 suicides per 100,000 residents during the same period (95% CI = 0.943 to 0.995, *p* = 0.020).

We should note that although the population of Western Crete (227,407) is considerably smaller compared to Eastern Crete (366,961), the availability of inpatient beds for psychiatric care in general hospitals is considerably higher in the former region than in the latter (15/100,000 vs. 9/100,000, respectively). A similar trend is noted for outpatient facilities available to support psychosocial rehabilitation (70/100,000 vs. 7/100,000, respectively) (https://www.hc-crete.gr/ψυχικη-υγεια/χαρτης-ψυχικης-υγειας).

## Discussion

This is the first long-term (1999–2013) study of suicides in the island of Crete based on data from the Department of Forensic Medicine of the University of Crete. The primary findings of the study are: (1) Crete has overall and yearly, higher SMRs compared to the national SMR in Greece, (2) there is no overall increase of SMRs including the period of the economic crisis (2008–2013), (3) there were opposing trends between men and women, with women showing a decrease, whereas men displaying increased rates in that period, (4) in men, there was a significant increase in middle-aged and elderly but not in the younger persons, although the highest unemployment rates were observed in young men and women and (5) SMRs have decreased in Western Crete and increased in Eastern Crete, an area where the network of mental health services is markedly underdeveloped.

Our data clearly indicate that the island of Crete in this 15-year period has both yearly and overall higher SMRs compared both to the national average SMR, as well as to the SMR of other parts of Greece, a phenomenon that is difficult to explain [13]. There is a popular belief that Crete has higher rates of psychopathology compared to the rest of Greece, but there are no epidemiological data to support such a claim. Well-established differences in terms of culture, such as large, close-knit families, high frequency of gun ownership and limited access to mental health services due to geographical factors and scarcity of such services may explain the higher SMRs in the island of Crete.

A major finding of our study is that the overall SMRs in Crete have remained stable across 15 years including the economic crisis period. This counters popular beliefs and previous study findings claiming an increase of suicides driven by the economic crisis. In 2011, a brief report in Lancet suggested that SMRs rose by 17% between 2007 and 2009 with a further 40% rise in the first half of 2011 as compared with the same period in 2010 [5]. Following this initial report, several studies reported modest, non-significant increases [3] based on unofficial sources, such as police announcements and news coverage [6], or even a reduction in suicides in 2012 [14]. Notably these studies contrasted short periods before and during the initial period of the crisis [3, 5]. A study based on ELSTAT data reported an increase in SMRs by 18% in men during the 2009–2012 period as compared to the 2005–2008 period, with relative stable rates among women [15]. Compared to these earlier studies, our study covers a much longer time period (including nine years prior to and six years during the crisis). This feature is important in light of the well-known yearly fluctuation of SMRs. For instance, the by-far highest suicide rate in Crete during the present study period occurred in 2000, a year marked by a steady rise of both national and regional gross income. Consistent with our study, Fountoulakis et al. reported no increase in SMRs in the area of Thessaloniki in Northern Greece for the same twelve year period (2000–2012) [7].

An interesting finding of our study is the age- and gender-dependence of SMRs. There was a significant increase of SMRs in middle aged men whereas no such association was observed in younger men (< 40 years old), despite the highest unemployment rates observed in this group (> 60%) (http://www.statistics.gr/en/home/). Similarly to our findings, a study in Italy reported decline of SMRs among younger men and increase among men involved in the labor force during the economic crisis compared to the period before [16]. One explanation of these findings is that a young man who is unemployed can either rely on the support of his family, a very common phenomenon in the Cretan culture indicating family members’ strong adherence to the family bonds, or can choose to emigrate in search of better job opportunities. Based on national statistics it is estimated that between 2008 and 2016, 300,000 young Greeks went abroad to find better job opportunities, the so called “brain-drain” phenomenon (http://www.bankofgreece.gr/BogEkdoseis/ekthdkth2015.pdf; http://www.kathimerini.gr/865921/article/epikairothta/ellada/to-trito-metanasteytiko-kyma-twn-ellhnwn) [17]. In contrast, middle-aged men traditionally carry the main burden of the economic well-being of the family. Thus, losing their job or business can have a devastating effect both on the socio-economic status of their families as well as on their self-esteem. Rising SMRs among elderly men in Greece, unlike findings from Italy [16] may be related to significant reductions in already modest pensions enforced as part of the austerity measures in our country. In women we observed a decreasing trend of SMRs during the period of the economic crisis, consistent with other studies conducted in Greece and other southern European countries [14–16]. A possible explanation is that during stressful periods women revert to their more traditional role of supporting their entire family at a social and personal level.

A recent 30-year interrupted time series analysis on the influence of austerity-related and prosperity-related events on SMRs in Greece during the period 1983–2012 found a significant increase in total suicides by 36%, and male suicides by 19%, after the introduction of new austerity measures in June 2011 [2] and in women an abrupt and sustained increase by 36% following austerity-related events in May 2011. Interestingly, during one prosperity-related event, i.e. the January 2002 launch of the Euro in Greece, there was an abrupt but temporary significant decrease in male suicides [2].

An important and novel finding of our study is that regional SMRs vary systematically with the amount of available mental health services. The results from the literature are mixed. One study in Greece reported a positive association between mental health services and suicide mortality rates. [18] However, another recent study including 10 European countries failed to find significant associations between available psychiatric beds and SMRs, after controlling for economic variables [19]. In our study, there was a significant decrease of suicides during this 15-year period in Western Crete, whereas there was a significant increase in Eastern Crete. Traditionally and for historical reasons, both inpatient and outpatient services have been more strongly developed in the western part of the island although the population in the eastern part of the island comprises 2/3 of the total population of Crete. Notably, in 2014 all parties of the Greek parliament recognized that mental health services were grossly underdeveloped in the eastern part of Crete [17] and that there is an urgent need to support and to expand these services in that region.

### Strengths and limitations

There are several strengths and limitations associated with this study. This is the longest –ever period studied for SMRs in Greece covering nine years preceding and six years after the economic crisis. Also, this study is based on data from the Forensic Medicine Department of the University of Crete, whereas suicides may be underreported in official national statistics due to family or social pressures to attribute death from suicide to other causes as a result of religious and cultural beliefs [9–11]. One limitation of our study is the relatively small sample size that might have affected the statistical significance of some of our findings, such as the gender effect. A further limitation of our study is that unemployment was the only indicator used to examine the impact of the crisis on economy, while others such as Per Capita Income, Household Income, home ownership etc. were not included. Finally, in interpreting the results of this study, we should consider some cultural and economic characteristics of the island of Crete. For example, the island is well-known for the close relationships of the members of the nuclear and extended family. Moreover, the burden of the economic crisis may have been contained in Crete by the fact that many residents benefit from additional sources of income (tourism and farming).

## Conclusions

In conclusion, our study indicates that in the island of Crete, despite overall stable SMRs during the economic crisis period, it appears that middle-aged and elderly men may be more vulnerable to the burden of economic turmoil and should be specifically targeted for appropriate prevention services. For example, “crisis lines” can be of particular value in the workplace to support middle-aged men who lose their jobs are laid off, supplemented by hot lines operated by “business associations”. Furthermore, our study strongly supports the usefulness of mental health services in preventing suicides during the years of economic crisis. In this regard, the delay in developing these services in Eastern Crete as a result of fiscal austerity and lack of available funds, can have a perpetuating and lasting effect on the observed rise of suicides in this part of the island. Finally, the decreasing trends of SMRs in younger men and in women, suggests that in this region, traditional values, such as close family and social ties, and lower social expectations may have a protective role against suicides in these age and gender groups. In this vein, advancing our understanding of those aspects of family and social life that may serve as protective factors against suicide risk is critical to develop prevention strategies.

## Abbreviations

ELSTAT: Hellenic statistical authority
SMR: Suicide mortality rate
